# One-Year Outcome of Multiple Blood–Brain Barrier Disruptions With Temozolomide for the Treatment of Glioblastoma

**DOI:** 10.3389/fonc.2020.01663

**Published:** 2020-09-10

**Authors:** So Hee Park, Myung Ji Kim, Hyun Ho Jung, Won Seok Chang, Hyun Seok Choi, Itay Rachmilevitch, Eyal Zadicario, Jin Woo Chang

**Affiliations:** ^1^Department of Neurosurgery, Brain Research Institute, Yonsei University College of Medicine, Seoul, South Korea; ^2^Department of Radiology, Yonsei University College of Medicine, Seoul, South Korea; ^3^InSightec, Ltd., Haifa, Israel

**Keywords:** glioblastoma multiforme, progression-free survival, blood–brain barrier, focused ultrasound, magnetic resonance images

## Abstract

**Introduction:** To overcome the blood–brain barrier (BBB) which interferes with the effect of chemotherapeutic agents, we performed multiple disruptions of BBB (BBBD) with magnetic resonance-guided focused ultrasound on patients with glioblastoma (GBM) during standard adjuvant temozolomide (TMZ) chemotherapy [clinical trial registration no.NCT03712293 (clinicaltrials.gov)]. We report a 1-year follow-up result of BBBD with TMZ for GBM.

**Methods:** From September 2018 to January 2019, six patients were enrolled (four men and two women, median age: 53 years, range: 50–67 years). Of the six patients, five underwent a total of six cycles of BBBD during standard TMZ adjuvant therapy. One patient underwent three cycles of BBBD but continued with TMZ chemotherapy. The 1-year follow-up results of these six patients were reviewed.

**Results:** The mean follow-up duration was 15.17 ± 1.72 months. Two patients showed a recurrence of tumor at 11 and 16 months, respectively. One underwent surgery, and the other patient was restarted with TMZ chemotherapy due to the tumor location with a highly possibility of surgical complications. The survival rate up to 1 year was 100%, and the other four patients are on observation without recurrence. None of the six patients had immediate or delayed BBBD-related complications.

**Conclusion:** Multiple BBBDs can be regarded as a safe procedure without long-term complications, and it seems to have some survival benefits. However, since TMZ partially crosses the BBB, a further extended study with large numbers would be needed to evaluate the benefits of BBBD resulting in an increase of TMZ concentration. This study opened a new therapeutic strategy for GBM by combining BBBD with a larger molecular agent.

## Introduction

Glioblastoma (GBM) is one of the most common and malignant primary brain tumors, making up 54% of all gliomas and 16% of all primary brain tumors (1). Its survival rate is low due to its aggressive nature in spite of the numerous efforts to overcome GBM with chemotherapeutic agents. Despite the use of standard treatment regimens since the 2000s, the median survival still remains to be 14 to 15 months only (2, 3).

One of the main reasons for this ineffectiveness of the chemotherapeutic agents is the presence of the blood–brain barrier (BBB). The BBB mechanically and biochemically restricts the passage of molecules. Furthermore, the peritumoral area is commonly infiltrated with tumor cells, but the BBB of this lesion is mostly intact. The infiltrative nature of GBM makes additional difficulties for the delivery of chemotherapeutic agents into the aimed targets (4).

Tumor treating fields (TTF) lead to tumor cell death or arrest, hypothetically by disrupting mitotic spindle formation and cell division with alternating electric fields to the tumor. The TTF was introduced in a pilot clinical trial for glial tumor in 2007 as a new treatment modality that is not affected by the presence of the BBB (5). A clinical trial showed some survival benefits of TTF on newly diagnosed GBM, whereas it did not show any benefit on recurrent tumors. Thus, some neuro-oncologists and neurosurgeons are still skeptical about TTF (6).

Recently, magnetic resonance-guided focused ultrasound (MRgFUS) with low-intensity energy has been attracting attention clinically as a non-invasive means of temporarily disrupting the BBB. Since it has been found that the transcranial delivery of ultrasound into the brain is feasible and low-intensity ultrasound can temporarily open the BBB, many preclinical studies have been conducted (7, 8). In the preclinical studies, the survival rate of the animal model for glial tumor or metastatic brain tumor increased when the drug was delivered with BBB disruption (BBBD) (9, 10). Since then, many clinical studies have been conducted with MRgFUS as a new modality to overcome the BBB (11–13). With its safety and feasibility confirmed, we tried to disrupt the BBB to improve the therapeutic effect in GBM during six cycles of standard temozolomide (TMZ) chemotherapy period for the first time in the world (13). We hereby report the 1-year follow-up results of BBBD with MRgFUS on GBM.

## Materials and Methods

**Figure 1:**
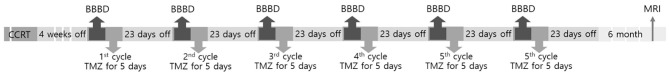
Overview of the study. CCRT, concurrent chemoradiation therapy; BBBD, blood–brain barrier disruption; TMZ, temozolomide; MRI, magnetic resonance imaging.

Six patients were enrolled from September 2018 to January 2019. The patients who underwent grossly total surgical resection (GTR) of tumor confirmed as grade IV malignant glioma by a neuropathologist were included. The patients received BBBD during the six cycles of chemotherapy of the standard TMZ treatment regimen. One cycle was defined as 28 days, and a total of six cycles were applied in the TMZ regimen of this study. On the first cycle, 150 mg/m^2^ of TMZ per day was taken for the first 5 days (days 1–5). On the following 2nd to 6th cycles, a dose of 200 mg/m^2^ was taken as maintenance dosage for the first 5 days of each cycle. BBBD was performed on the first or the second day of the 4-week chemotherapy cycle (Figure 1).

The BBBD target was selected within 2 cm of the tumor margin, mainly on white matter with high signal intensity on the fluid attenuated inversion recovery images. An average of 4.4 ± 0.9 BBBD targets was selected per patient without overlapping margin by a neurosurgeon and a neuroradiologist with a pre-sonication MRI. The BBBD was performed on 1 cm^3^ per target and on the same targets for every cycle. BBBD was performed using the ExAblate low-frequency MRgFUS system (ExAblate Neuro Model 4000 Type 2.0 220 kHz system, InSightec, Haifa, Israel). MRgFUS was performed with an intraoperative MRI based on the stereotactic frame. Ultrasound was delivered with an intravenous injection of microbubble. The bubble activity was monitored in real-time during sonication. Sonication was performed for about 210 s per target.

MRI was performed before and after each BBBD treatment and at 6 months after the 6th cycle of chemotherapy. The feasibility of BBB was determined by comparing T1-enhanced or T2^*^/GRE MRI before and immediately after the procedure. All performed MRI were used to identify the immediate or the delayed radiological adverse events and to assess tumor status according to the Response Assessment in Neuro-Oncology criteria. This study was approved by our institutional review board (1-2018-0040), and patient's informed consent was obtained [clinical trial registration no. NCT03712293 (clinicaltrials.gov)].

Five of the six patients underwent a total of six cycles of BBBD during standard TMZ adjuvant therapy. One patient dropped out after three sessions of BBBD, but the patient still continued with the TMZ chemotherapy. The 1-year follow-up results focusing on the outcome and the complications of these six patients were reviewed.

## Results

Six patients (four men and two women, median age: 53 years, range: 50–67 years) were followed up for an average of 12.17 ± 1.94 months from the beginning of their first BBBD. It is on average 15.17 months from the initial diagnosis of GBM (minimum 13 months, maximum 18 months). The demographics of the patients who underwent BBBD are shown in Table 1. BBBD was confirmed by post-sonication MRI in the median of 94.3% of the target (minimum 70.8%, maximum 100%) during the six cycles of BBBD (13).

**Table 1:** Patients demographics.

	**Age**	**Sex**	**Location**	**IDH**	***MGMT* methylation**	**1p19q codeletion**	**EGFR**	**Neurological status**
Patient 1	53	F	Lt.F	Wild	+	–	–	No deficit
Patient 2	62	M	Rt.T	Wild	+	–	–	No deficit
Patient 3	53	M	Rt.P	Wild	–	–	–	No deficit
Patient 4	67	M	Lt.T	Wild	+	–	–	No deficit
Patient 5	50	M	Lt.P	Wild	+	–	–	No deficit
Patient 6	50	F	Rt.P	Wild	–	–	–	No deficit

*IDH, Isocitrate dehydrogenase; MGMT, Methylguanine–DNA methyltransferase promoter; EGFR, Epidermal growth factor receptor*.

**Figure 2:**
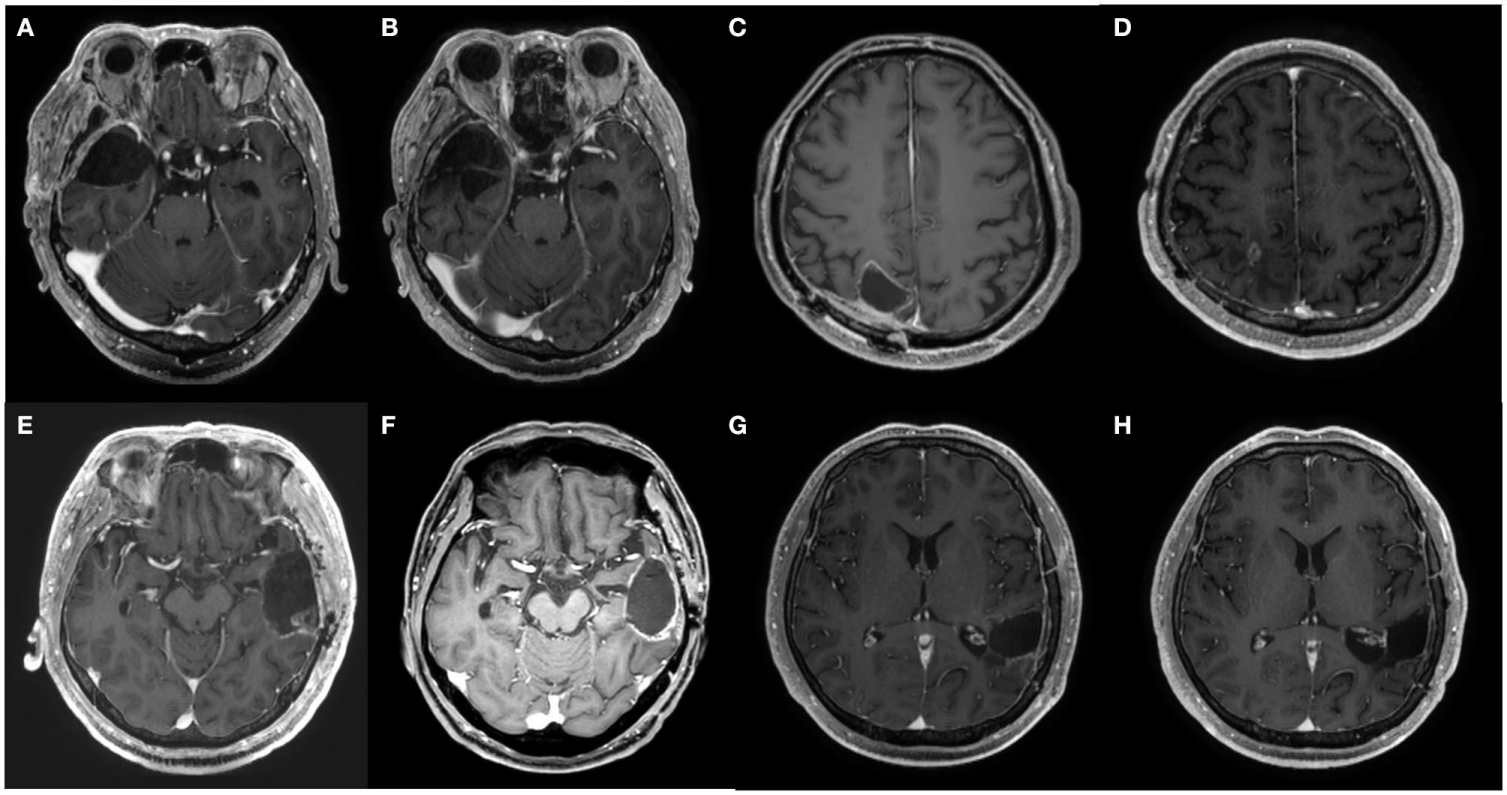
Axial MRI images at pretreatment and at 1 year after the chemotherapy of patients 2, 3, 4, and 5. **(A,B)** Pre- and post-treatment MRI of patient 2, respectively. **(C,D)** Pre- and post-treatment MRI of patient 3, respectively. **(E,F)** Pre- and post-treatment MRI of patient 4, respectively. **(G,H)** Pre- and post-treatment MRI of patient 5, respectively.

From the more than 1 year of follow-up period, two patients showed recurrence at 11 and 16 months of follow-up, respectively. The other four patients showed no recurrence for an average of 15 months (Figure 2). The survival rate up to 13 months was 100%. None of the six patients had immediate or delayed BBBD-related complications (Table 2).

**Table 2:** The results of six patients who underwent BBBD.

	**Follow-up period (months)**	**Survival**	**Recurrence**	**MRgFUS related complications**
Patient 1	18	+	+	–
Patient 2	15	+	–	–
Patient 3	16	+	–	–
Patient 4	15	+	–	–
Patient 5	14	+	–	–
Patient 6	13	+	+	–

*BBBD, blood-brain barrier disruption; MRgFUS, Magnetic resonance guided focused ultrasound*.

## Case Description

### Patient 1

**Figure 3:**
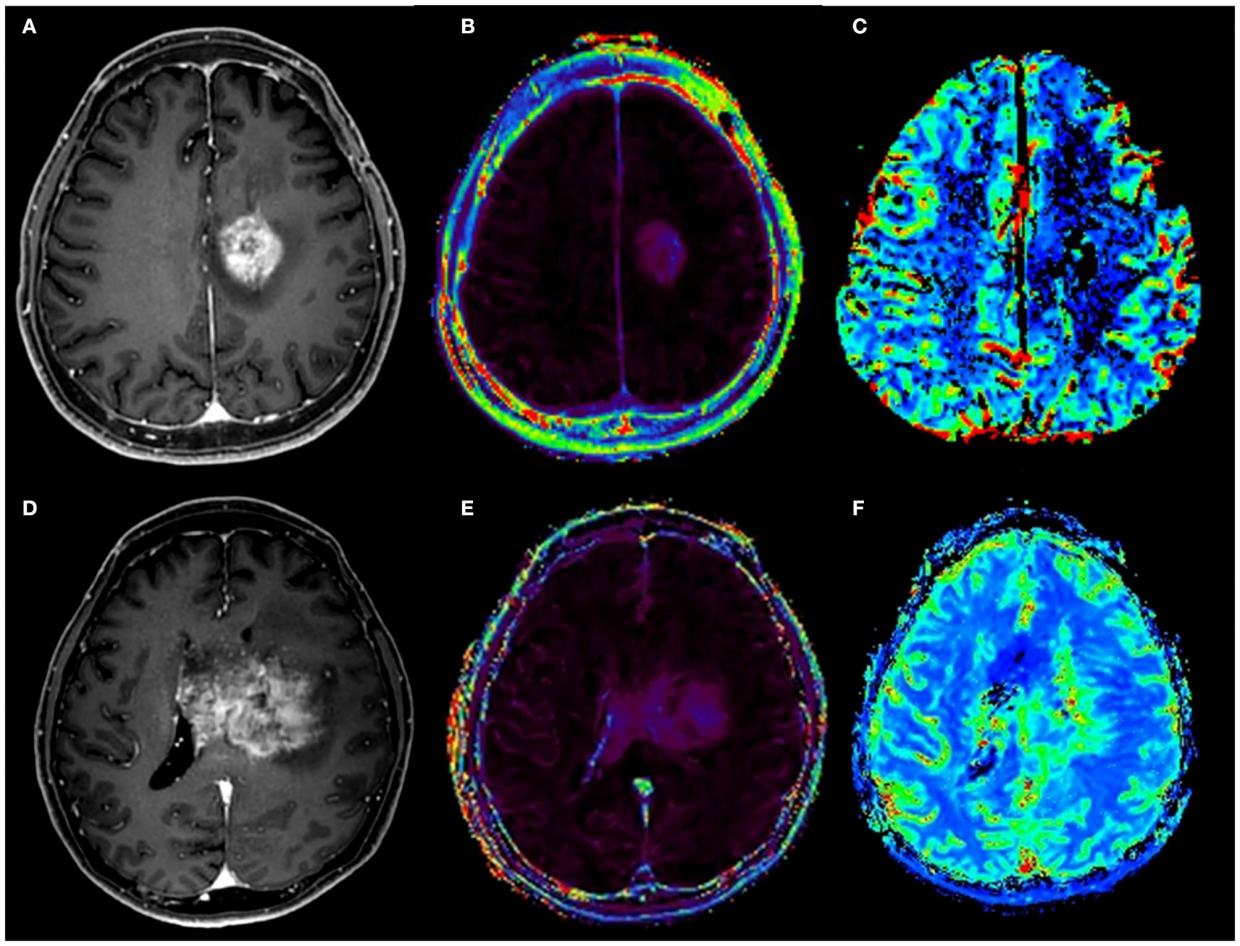
Pseudoprogression and recurrence of glioblastoma (GBM) in patient 1 after six cycles of adjuvant temozolomide (TMZ) chemotherapy and blood–brain barrier disruption. After the six cycles of adjuvant TMZ chemotherapy, a newly developed enhanced lesion at the left cingulate gyrus was observed on contrast-enhanced T1-weighted MRI **(A)**. However, relative cerebral blood volume **(B)** and volume transfer coefficient (Ktrans) **(C)** maps showed no increase of metrics, suggesting pseudoprogression. At 4 months later, MRI was performed due to the reappearance of symptoms. On contrast-enhanced T1-weighted MRI **(D)**, a larger enhanced mass with elevated Ktrans **(E)** and increased cerebral blood volume **(F)** are shown.

Two months after the completion of all six cycles of BBBD, patient 1 demonstrated a mild motor weakness of the right leg. Pseudoprogression (PsP) of the tumor at the site of the BBBD was observed on MRI. The neurological symptom disappeared after steroid therapy. On the 3-month follow-up MRI, the enhanced lesion showed a decrease in size (Figure 3). The symptoms reappeared over time and a follow-up MRI showed the recurrence of GBM at 16 months from its first diagnosis, i.e., 8 months from the completion of BBBD (Figure 3). The patient was restarted with TMZ chemotherapy because the surgical resection had a high possibility of complication due to the location of the tumor mass. The patient is currently under observation, with right side motor weakness.

### Patients 2, 3, and 5

The patients completed six cycles of chemotherapy and BBBD without any adverse events or recurrence of the tumor and have been observed as in a clinically and radiologically stable disease state.

### Patient 4

The patient had a GBM on the left temporal area and demonstrated a minor personality issue before enrollment. After three cycles of BBBD, the patient dropped out from the study. However, he still continued on the TMZ chemotherapy for the remaining three cycles. He has been observed as in a stable disease state without any findings of recurrence on the MRI performed at 6 months after the completion of chemotherapy and clinical evaluation.

### Patient 6

**Figure 4:**
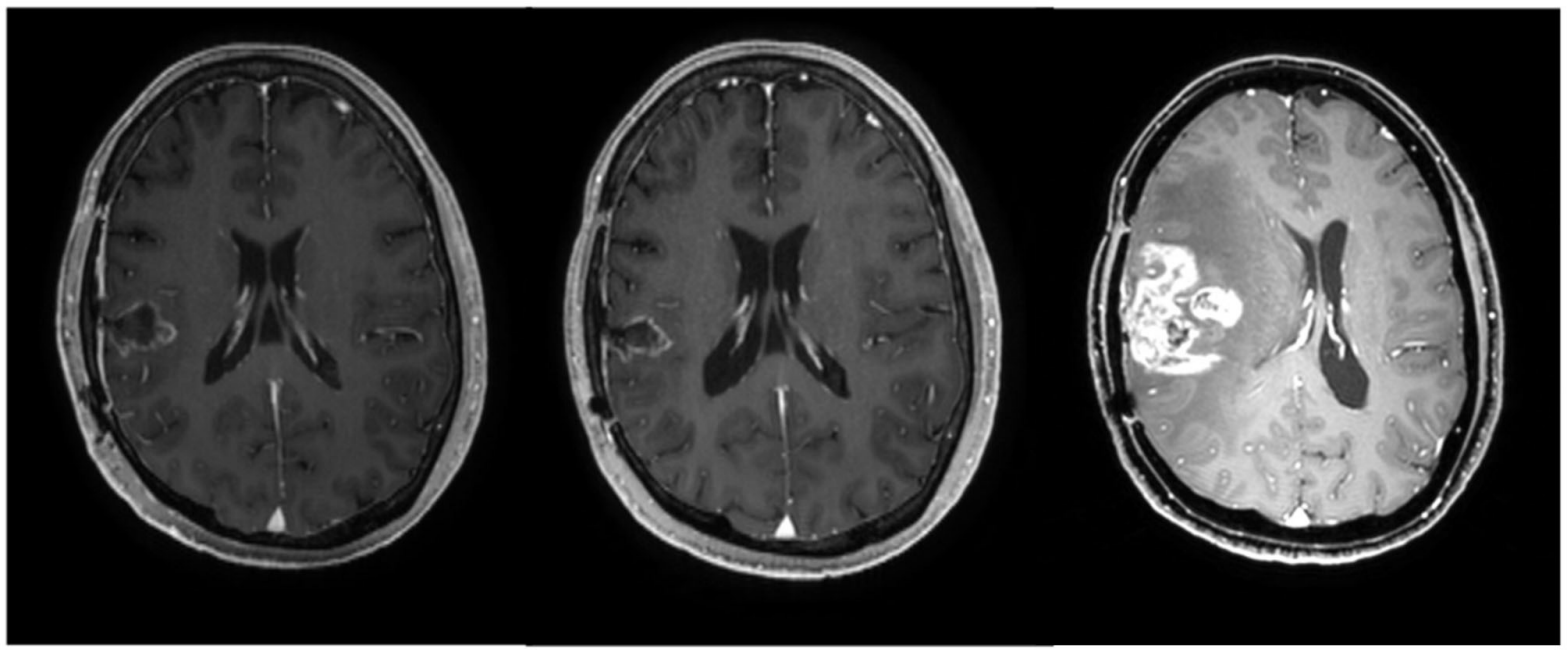
Axial MRI images of patient 6 at 3 months (before chemotherapy), 9 months (after the completion of chemotherapy), and 11 months after the initial diagnosis of glioblastoma, respectively.

After completion of the chemotherapy, the patient had no neurological deficit and showed a stable disease state without evidence of recurrence on MRI. However, at 2 months later, i.e., 11 months from the initial diagnosis of GBM, the patient complained of left motor weakness and a recurrence at the right temporal lobe was observed on follow-up MRI (Figure 4). Surgical resection was done and chemotherapy is in progress.

## Discussion

GBM is one of the most common but also one of the most lethal malignant brain tumors. It occurs in the cortex, grows invasively and aggressively, invades the lobes, and affects the deep structures of the brain (14). Due to these characteristics, patients with GBM have short recurrence-free interval, short survival time, and poor prognosis. Prior to the release of the currently used drug, TMZ, its treatment consisted of surgical resection followed by radiation therapy, yielding a median survival between 9 to 12 months (15–17).

Brain tumors are not well-controlled with therapeutic agents because of the BBB. The BBB, a barrier formed by tight junctions of endothelial cells, imposes size, and biochemical restrictions on molecule passage. Therefore, large and hydrophilic molecules cannot pass through the BBB, whereas small lipid-soluble drugs below 400 Da can (18). The TMZ molecule is 194 Da in size and lipophilic, available for better central nervous system penetration compared to other alkylating agents (19). Due to these characteristics, the present standard treatment regimen for GBM is surgery, radiotherapy, and chemotherapy of concomitant and six cycles of adjuvant TMZ (20). After the introduction of TMZ and the currently used regimen, the median survival has increased up to 14.6 months, the 2-year survival rate up to 27.2%, and the 5-year survival up to 10% (20, 21).

Many efforts have been made to increase the survival rates, but they have been unsuccessful. Changing the dose frequency or delivery method, such as intra-arterial delivery with chemotherapeutic agents, resulted in higher toxicity rates without improving patient outcome (22, 23). In addition, spatially non-specific methods, such as convection-enhanced delivery or wafer, had adverse effects on normal brain tissue (4). Recently, several studies have shown that a very low intensity of ultrasound can disrupt the BBB without damaging the surrounding brain tissues (8, 11). Since then, many studies have been conducted using MRgFUS as a non-invasive means of temporarily disrupting the BBB.

Although TMZ can pass through the BBB, the concentration of TMZ in brain tumor tissue is about 20% of the plasma level (19). Even with the concurrent radiation therapy which is performed to improve the BBB penetration of TMZ, the concentration may rise only up to 35% of the plasma level (24). A previous study demonstrated that BBBD has made a 7.7-fold difference of TMZ concentration between the sonicated and the unsonicated areas of a patient (12). Therefore, we tried to increase the effect of tumor control by BBBD using MRgFUS.

Previous studies postulate that GBM generally recurs at a median of 7–7.5 months after a primary treatment and has an average survival of 12.8–14.6 months (25, 26). A recent meta-analysis showed that the progression-free survival (PFS) is 66.5% at 6 months and 46.8% at 1 year, with 55.9% of 1-year overall survival rate when total gross removal is performed for GBM (27). When GBM is subtotally removed, this is lowered to 51.3, 23.3, and 39.8%, respectively. All six patients in this study underwent GTR of tumor. Although two of the six patients had a recurrence, they showed 100% of 6-month PFS and 83.3% of 1-year PFS, which is better than the previously reported meta-analysis. The average PFS of the patients who had a recurrence was 13.5 months, whereas the others showed a median PFS of at least 15 months. This is twice the previously reported PFS and is likely to increase. In addition, the 1-year overall survival was 100%. Regarding these results, the survival benefit of additional BBBD to increase TMZ concentration may be considered.

One of the factors affecting the effect of TMZ is the presence of O6-methylguanine-DNA methyltransferase (MGMT) gene methylation. The MGMT is a DNA repair protein. The methylation of *MGMT* promoter is associated with the loss of MGMT expression, diminished DNA repairing activity induced by alkylating agents, and longer survival (28, 29). Of the four patients without progression during the follow-up period, three patients had *MGMT* methylated status. In addition, of the patients with recurrent GBM, one patient with methylated *MGMT* promoter had a recurrence at 16 months and the other patient with unmethylated *MGMT* promoter had a recurrence at 11 months after the initial diagnosis. Thus, even with BBBD, patients with methylated *MGMT* promoter seem to show a better clinical course and treatment results despite the fact that it is difficult to identify the significant correlation between *MGMT* promoter methylation and recurrence or survival due to the small size of the study. However, our study results show that 75% of patients (3/4) with methylated *MGMT* promoter had no evidence of recurrence for an average of 14.7 months, whereas 50% of patients (1/2) with unmethylated *MGMT* promoter had no recurrence for an average of 16 months. Therefore, the increased survival benefits of elevated TMZ concentration due to BBBD in patients with unmethylated *MGMT* promoter should be emphasized.

Despite that the results of multiple BBBDs in addition to TMZ have been positive to date with survival benefit, a more careful approach is needed to evaluate the benefits of BBBD, resulting in an increase of TMZ concentration. A long-term follow-up study beyond the commonly reported median survival period and a further extended study with a larger number would be needed to determine how positively BBBD affects the survival period of GBM.

There were no short-term or long-term complications associated with BBBD during the 15 months of median follow-up period in this study. Many studies have reported that BBBD with MRgFUS is safe without complications (11, 12). It has also been reported that multiple BBBDs have been performed without short-term complications (13). This study confirmed that the long-term follow-up result of multiple BBBDs is safe and without complications such as radiological adverse events or TMZ-related neurotoxicity or clinically adverse events.

In one patient, PsP was observed at the site of the BBBD after six cycles of adjuvant TMZ chemotherapy. The PsP is a subacute treatment-related effect, which usually occurs within 3 months of completion of chemoradiation in 15 to 30% of patients (30, 31). It likely reflects an inflammatory post-procedure tissue reaction after a successful local treatment, followed by long-lasting tumor control. The presence of *MGMT* promoter methylation is associated with PsP (30). A patient with PsP in this study had *MGMT* methylated status. However, it took 8 months before PsP was observed after the completion of chemoradiation, whereas it usually occurs within 3 months after chemoradiation. Recurrence was noted afterwards. There are several possibilities on whether PsP occurs as a local inflammation related to tumor control of BBBD or is caused by BBBD regardless of the underlying disease and whether BBBD affects the timing of PsP or recurrence. Therefore, additional follow-up will be needed and further research on the relationship and the mechanism between BBBD and PsP would be required.

In conclusion, multiple BBBDs can be regarded as a safe procedure without long-term complication, and it seems to have some survival benefits. This study opened a new therapeutic strategy for GBM by combining BBBD with a therapeutic agent which could not be used due to BBB.

## Data Availability Statement

The raw data supporting the conclusions of this article will be made available by the authors, without undue reservation.

## Ethics Statement

The studies involving human participants were reviewed and approved by severance hospital institutional review board 1-2018-0040. The patients/participants provided their written informed consent to participate in this study. Written informed consent was obtained from the individual(s) for the publication of any potentially identifiable images or data included in this article.

## Author Contributions

JC and EZ contributed to the conceptualization of the study and the development of the methodology. SP, MK, HC, and IR performed formal analysis and investigation. SP wrote the original draft preparation. JC, HJ, and WC wrote, reviewed, and edited the manuscript. JC took charge of funding acquisition and supervised the study. All authors contributed to the article and approved the submitted version.

## Conflict of Interest

IR and EZ are employees of InSightec. The remaining authors declare that the research was conducted in the absence of any commercial or financial relationships that could be construed as a potential conflict of interest.
